# Development and Assessment of Novel Predictive Nomograms Based on APRI for Hepatitis B Virus-associated Small Solitary Hepatocellular Carcinoma with Stereotactic Body Radiotherapy

**DOI:** 10.7150/jca.47291

**Published:** 2020-09-23

**Authors:** Lin Lai, Tingshi Su, Zhongguo Liang, Yunxing Lu, Encun Hou, Zuping Lian, Hongjun Gao, Xiaodong Zhu

**Affiliations:** 1 Department of Oncology, Wuming Hospital of Guangxi Medical University, Nanning 530199, Guangxi, People's Republic of China.; 2 Department of Radiotherapy, Guangxi Medical University Cancer Hospital, Nanning 530021, Guangxi, People's Republic of China.; 3 Department of Medical Oncology, Ruikang Hospital Affiliated to Guangxi Traditional Chinese Medical University, Nanning 530011, Guangxi, People's Republic of China.; 4 Department of Urology, Ruikang Hospital Affiliated to Guangxi Traditional Chinese Medical University, Nanning 530011, Guangxi, People's Republic of China.

**Keywords:** hepatocellular carcinoma, aspartate aminotransferase-to-platelet index, stereotactic body radiotherapy

## Abstract

**Background:** The correlation between serum inflammatory marker before treatment and the survival of patients with hepatitis B virus (HBV)-associated small solitary hepatocellular carcinoma (HCC) after stereotactic body radiotherapy (SBRT) remains unclear. The objective of our study is to estimate survival in such patients using multivariable prediction models and investigate the prognostic value of aspartate aminotransferase-to-platelet index (APRI), neutrophil-to-lymphocyte ratio (NLR), platelet-to-lymphocyte ratio (PLR), and lymphocyte-to-monocyte ratio (LMR) for HBV-associated small solitary HCC patients treated with SBRT.

**Patients and methods:** Patients with HBV-associated small solitary HCC who were newly treated with SBRT were retrospectively analysed in our hospital from 2009 to 2016. We counted the APRI, NLR, PLR, and LMR before treatment and calculated their cut-off values for predicting overall survival (OS) and progression-free survival (PFS) by receiver operating characteristic (ROC) analysis. The random forest model combined with least absolute shrinkage and selection operator (LASSO) regression model for OS and PFS were used to screen potentially prognostic factors from serum inflammatory markers, demographic data, and clinical characteristics. Predictive models for OS and PFS were developed by multivariable COX regression and nomograms were constructed. Discrimination was assessed using the C-index. Internal validation was assessed using the Bootstrap method. Survival analysis was carried out to assess the prognostic value of serum inflammatory markers, and OS and PFS curves were compared by Kaplan-Meier analysis and Log-Rank test, respectively.

**Results:** A total of 72 patients with HBV-associated small solitary HCC were recruited for the study. The median follow-up time was 2015 days (range, 232-3823 days). Age, tumor size, NLR, PLR, and APRI were used to construct nomogram for OS, while gender, age, TNM stage, portal hypertension, AFP, APRI were for PFS. The two models displayed good discriminations with C-indexes of 0.738 (95% CI: 0.632-0.844) and 0.657 (95% CI: 0.538-0.777), and their C-indexes in the internal validation cohort reached 0.790 (95% CI: 0.684-0.896) and 0.739 (95% CI: 0.619-0.859). The multivariable cox analysis indicated that APRI<0.47 was favourable independent prognostic factors for OS and PFS. Compared to APRI≥0.47, APRI<0.47 predicts better OS (*p*=0.003) and PFS (*p*=0.003).

**Conclusions:** Nomograms based on APRI are superior in predicting OS and PFS in HBV-associated small solitary HCC patients who have received SBRT. APRI before treatment is a feasible and convenient prognostic indicator for OS and PFS, which helpfully determines the beneficial population of SBRT for HBV-associated small solitary HCC.

## Introduction

HCC is the sixth most common malignant tumor and the fourth leading cause of death globally^1^. When diagnosed with HCC, approximately 70% of cases are at later stages, leading a poor 5-year OS rate of 5%^2^. For early liver cancer, surgery and liver transplantation are the first choices 3, providing an excellent prognosis with a 5-year OS rate of around 40-70%^4^. However, the indication of hepatic resection has been limited to patients who are reluctant to undergo surgery or the aged under too high risk for surgery, and the application of liver transplantation is restricted because of insufficient liver donors^5^. Consequently, the consensus by the Asia-Pacific Primary Liver Cancer Expert (APPLE)^6^ and guidelines by the National Comprehensive Cancer Network (NCCN)^7^ all proposed that SBRT can be used as an optional radical treatment for early HCC, and our center has also carried out relevant reports^8^, ^9^. It is vitally important to identify patients that may most likely benefit from SBRT^10^.

China is home to approximately 51% HCC patients^11^. The vast majority of patients are complicated with HBV infection^12^. Chronic fibrosis and liver cirrhosis triggered by HBV not only are the main pathogenic risk factors but also have an important impact on the prognosis of liver cancer^13^. Serum inflammatory markers that monitor and evaluate systemic inflammatory responses can be easily calculated, and such parameters commonly used include APRI, NLR, PLR, and LMR and so forth 14, 15. The predictive role of serum inflammatory markers in cancer has been paid more and more attention. The prognostic value of serum inflammation indicators before treatment for liver cancer has been confirmed by studies focus on surgical resection, liver transplantation, target therapy, radiofrequency ablation (RFA),transcatheter arterial chemoembolization (TACE) and selective internal radiation therapy (SIRT)^16^-^21^. However, studies about cases of liver cancer treated with SBRT rarely reported prognostic inflammatory markers, instead, most of which merely focused on NLR and PLR^22^. This article hopes to develop visualized multivariate predictive nomograms and identify more possible novel prognostic indicators for SBRT therapy in hepatitis B-related small solitary HCC by reviewing the existing data.

## Material and Methods

### Patient population

Data from liver cancer patients who received SBRT were collected from January 1, 2009 to December 31, 2016 at Ruikang Hospital Affiliated to Guangxi Traditional Chinese Medical University. The enrolled patients met the following criteria: 1. small hepatocellular carcinoma patients with only one single nodule in the liver and a maximum diameter ≤50 mm without portal vein tumor thrombus, abdominal lymph nodes or distant metastasis; 2. all patients treated for the first time and with no previous tumor history; 3. following European Association for the Study of the Liver/European Organization for Research on Treatment of Cancer (EASL/EORTC) guidelines^23^, with definite pathological data or clinical diagnosis by liver enhanced CT and MRI before treatment; 4. before treatment, hepatitis B serum markers at least met HBsAg positive criteria; 5. All the included patients only received radical SBRT in the primary treatment without any other treatment before disease progression. The study protocol conforms to the Declaration of Helsinki and was approved by the Ethics Committee of Rui Kang Hospital Affiliated to Guangxi Traditional Chinese Medical University. However, due to the retrospective nature of the study, written informed consent was not required from the patients, and the research data were confidential.

### Laboratory data

Complete peripheral blood tests were carried out before SBRT treatment, including neutrophil count (NEUT), lymphocyte count (LYM), monocyte count (MONO), platelet count (PLT), aspartate aminotransferase (AST). The clinical diagnosis of portal hypertension was based on the definition in line with the American Association for the Study of Liver Diseases/European Association for the Study of the Liver (AASLD/EASL) guidelines^23^, ^24^, that is, the presence of endoscopic manifestations of oesophageal varicose veins or thrombocytopenia associated with splenomegaly with a platelet count <100×10^9^/L. The NEUT, LYM, MONO and PLT was tested by XE-5000 Automatic hematologic analyser (Sysmex Corp., Kobe, Japan), and AST was tested by AU2700 Automatic biochemical analyser (Beckman Coulter, Inc., Brea, USA). The calculation formulas of serum inflammatory markers are as follows:

NLR=Neutrophil Count (×10^9^/L) /Lymphocyte Count (×10^9^/L)

PLR= Platelet Count (×10^9^/L) /Lymphocyte Count (×10^9^/L)

LMR= Lymphocyte Count (×10^9^/L) /Monocyte Count (×10^9^/L)

APRI = [(AST value(U/L) /upper limit of normal value (U/L) /PLT (×10^9^/L)] × 100

### CyberKnife SBRT treatment

Before treatment, 3-4 gold markers with a diameter of 0.8 mm were implanted around the tumor tissue under the guidance of B ultrasound or CT. A week after the gold mark was implanted, CT and MRI scans were carried out to locate and describe the gross tumor target volume (GTV) and organ at risk (OAR) by fusing the reference images of CT plain scan and enhanced MRI scan (slice thickness 3 mm), and the planned target volume (PTV) was formed by expanding 0-3 mm of the GTV. The CyberKnife Synchrony respiratory tracking system (Accuray Inc., CA, USA) was used with a prescription dose of 36-48 Gy in 3-5 fractions on consecutive days, the 57%-80% isodose line (median 67%) covered the PTV. The biologically effective dose (BED) was calculated by the formula: D (1 + d/[α/β]) with D and d being the total dose delivered and dose per fraction, which is based on the value of α/β of 10 Gy (BED 10) 25.

### Follow-up

The re-examination items included laboratory indices and CT or MRI. Patients who were discharged from the hospital after SBRT treatment usually completed their first re-examination within 1-2 months, then once every 3 months, and once a year at 5 years and thereafter. According to the Modified Response Evaluation Criteria in Solid Tumor (mRECIST 1.1) standard, the presence or absence of disease progression were evaluated. Long-term efficacy evaluation included OS and PFS. OS was defined as the time from the SBRT start date to death or the end of follow-up date. PFS was defined as the time from the SBRT start date to the progression of the disease or the end of follow-up date.

### Statistical analysis

The optimal cut-off values of APRI, NLR, PLR, and LMR for predicting OS and PFS were determined by ROC curve analysis. All variables consisted of serum inflammatory markers, demographic data, and clinical characteristics, and a dimensionality reduction-based feature selection was carried out. Specifically, the relative importance of each variable was scored and sorted in a descending order using Random Forest prior to predictive features were selected using a LASSO regression model. Then, proposed predictive models for OS and PFS were developed using multivariate COX regression analysis and the corresponding nomograms were constructed. Harrell's C-index and AUC value were measured to quantify the discrimination performance of the nomograms. The predictive models were internally validated (1,0000 bootstrap resamples) using Bootstrap resampling to upgrade the models for higher accuracy, and the C-index values of upgraded models were calculated. Kaplan-Meier analysis and the log-rank test were applied to calculate the survival rate and compare the survival differences between patients with different levels of prognostic inflammatory marker. Spearman's rho test was applied to evaluate the correlations between the prognostic serum inflammatory factor and different clinical features. Statistical analysis was performed by R software package (v.3.6.1, https:// www.R-project.org) and SPSS Statistics Version 25.0 software (IBM Corp., Armonk, NY, USA). All statistical tests were two-sided, and *p<0.05 was* considered statistically significant.

## Results

### Patients characteristics

Seventy-two patients who met the inclusion criteria were finally enrolled, all of whom had CT or MRI as baseline data before treatment, with a median age of 57 years (range, 30-84 years). Men accounted for 85% and women accounted for 15%. Nineteen patients were confirmed by pathology, all of whom had hepatocellular carcinomas, and the rest were clinically confirmed. Fifteen cases of portal hypertension were clinically diagnosed, and fifty-seven cases had no portal hypertension. The laboratory values before treatment were as follows: median NEUT: 2.86×10^9^/L (range: 1.06-8.04×10^9^/L); median LYM: 1.51×10^9^/L (range: 1.06-8.04×10^9^/L); median MONO: 0.465×10^9^/L (range: 0.19-0.93×10^9^/L); median PLT: 160.5×10^9^/L (range: 31-355×10^9^/L); median AST: 29 U/L (range: 13-199 U/L); median APRI: 0.42(range: 0.12-14.27); median NLR: 1.88 (range: 0.56-5.08); median PLR: 94.36(range: 30.39-289.86); median LMR: 3.485(range: 1.4-6.94) (Table 1). No organ failure or complications with blood system or rheumatic immune system diseases were observed before treatment.

### Follow-up

All patients completed the first re-examination within 2 months after treatment and then returned to the hospital for re-examination once every 3-6 months. The patients received additional salvage regimens including TACE, RFA, drug and palliative care after disease progression. The survival information of all patients was obtained by retrieving medical records or telephone follow-ups. The follow-up deadline was January 31, 2020. The median follow-up time was 2015 days (range, 232-3823 days). As of the end date of follow-up, 23 patients were free of disease progression, 35 patients died and 37 patients survived.

### Inflammatory Markers and Cut-off Values

The ROC analysis showed that the optimal cut-off values of NLR in predicting OS and PFS were 1.87 and 1.89, with corresponding areas under the curve (AUCs) of 0.520 (95% CI, 0.385-0.655) and 0.546 (95% CI, 0.402-0.690), respectively. The optimal cut-off values of PLR for OS and PFS were 78.06 and 66.96, with AUCs of 0.405 (95% CI, 0.270-0.539) and 0.465 (95% CI, 0.311-0.619), respectively. The optimal cut-off values of LMR for OS and PFS were 3.16 and 2.44, with AUCs of 0.459 (95% CI, 0.324-0.593) and 0.483 (95% CI, 0.335-0.631), respectively. The optimal cut-off values of APRI in predicting OS and PFS were both 0.47, with AUCs of 0.690 (95% CI, 0.564-0.816) and 0.569 (95% CI, 0.431-0.707), respectively.

### Variables selection

**Figure 1:**
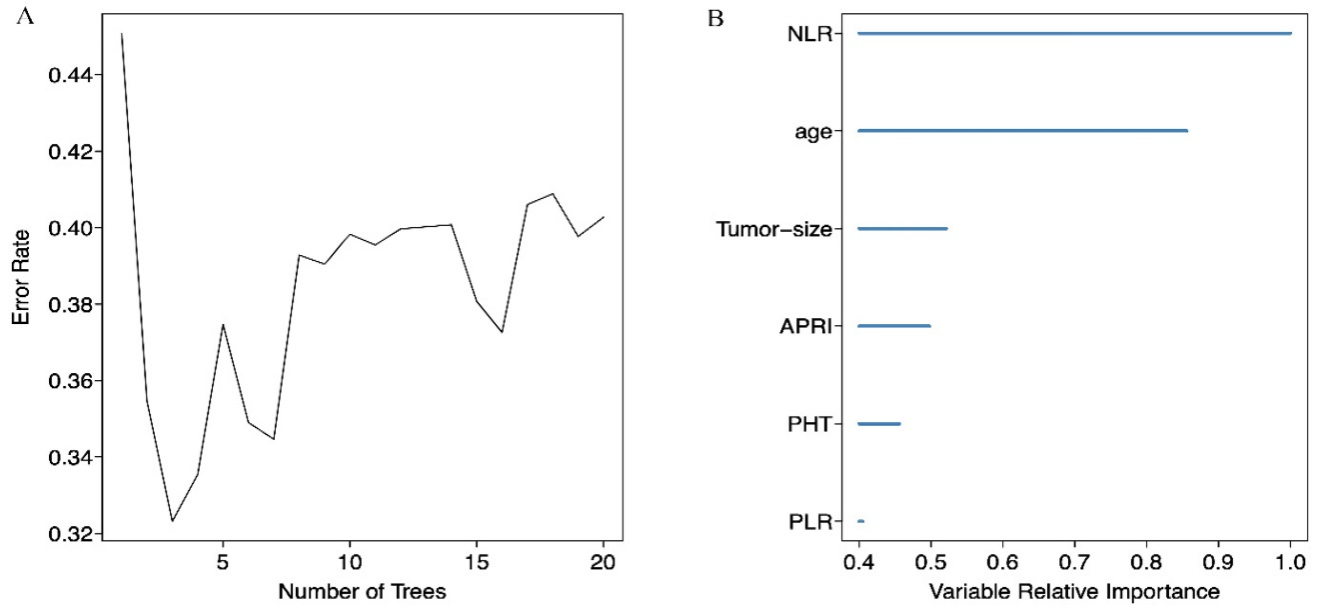
(A) Relationship between the error rate and the number of classification trees for OS; (B) The top 6 predictors for OS in order of importance.

**Figure 2:**
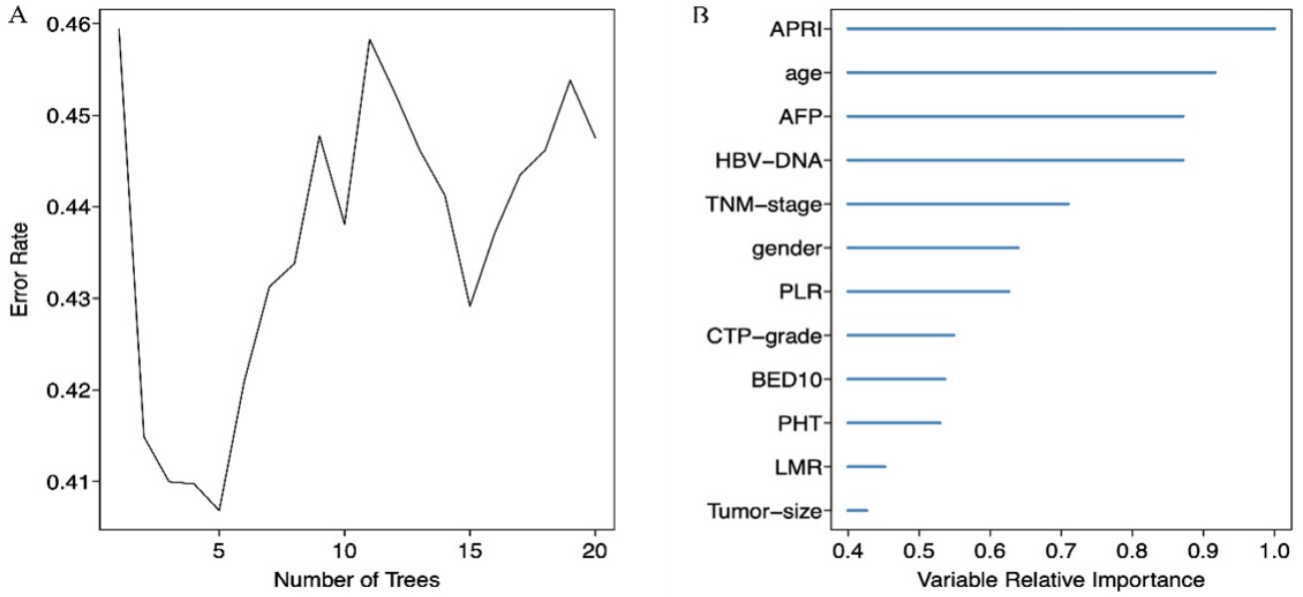
(A) Relationship between the error rate and the number of classification trees for PFS; (B) The top 12 predictors for PFS in order of importance.

We screened possible prognostic factors for OS and PFS from 13 variables including gender, age, HBV DNA, CTP grade, portal hypertension (PHT), tumor size, TNM stage, AFP, BED10, APRI, NLR, PLR, and LMR. The features were selected and ranked in a descending order of relative importance using random forest model. As a result, we identified the top 6 (NLR, age, tumor size, APRI, PHT, PLR) (Figure 1A, 1B) and 12 (APRI, age, AFP, HBVDNA, tumor size, gender, PLR, CTP grade, BED10, PHT, LMR, and TNM stage) (Figure 2A, 2B) predictors for OS and PFS in order of relative importance.

**Figure 3:**
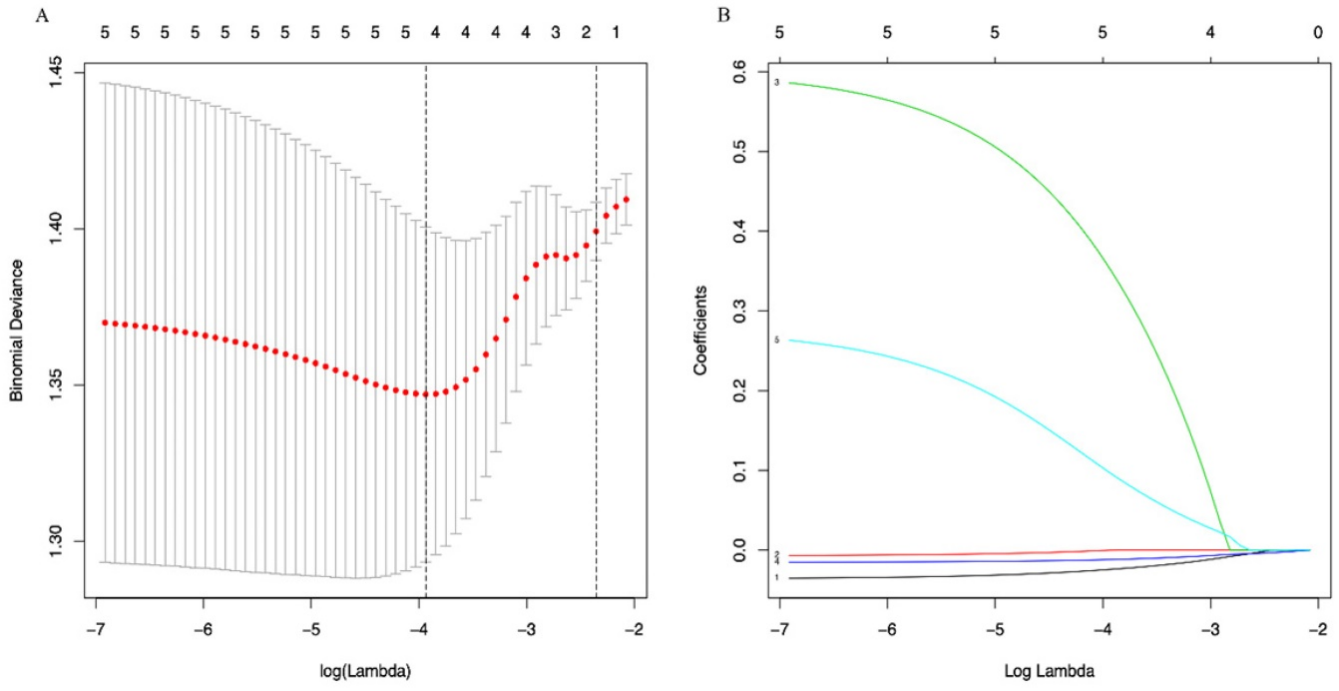
(A) Optimal (minimum) lambda selection for OS in the LASSO regression model; (B) LASSO coefficient profiles of variables selected for OS.

**Figure 4:**
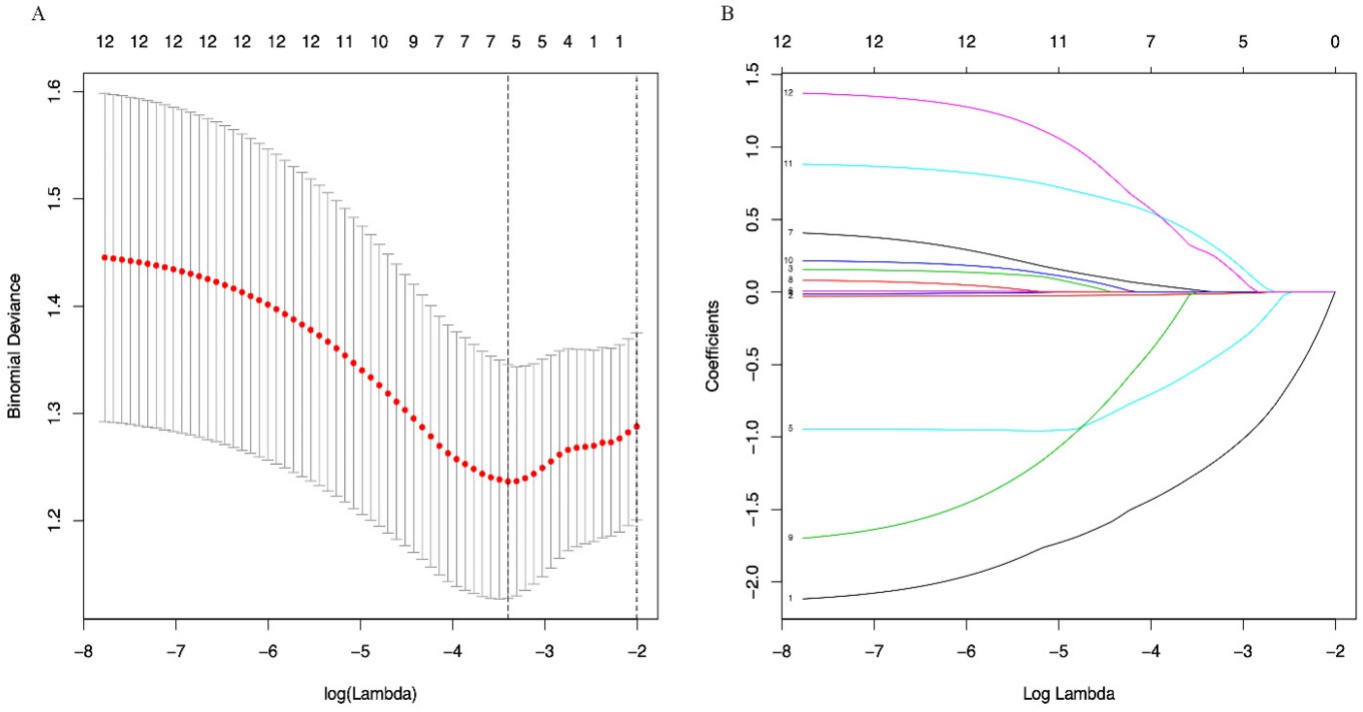
(A) Optimal (minimum) lambda selection for PFS in the LASSO regression model; (B) LASSO coefficient profiles of variables selected for PFS. Notes: Figure 3, 4 (A) The binomial deviance curve was plotted versus log(lambda). Dotted vertical lines were drawn at the optimal lambda values (0.01951751 for OS and 0.03333645 for PFS) using the minimum criteria; (B) A coefficient profile plot was produced against the log(lambda) sequence.

A LASSO regression model with 5-fold cross-validation was employed to select predictive variables among the preliminarily screened factors. Five and 6 features with nonzero coefficients that minimized the overall Wilk's Lambda were confirmed as the potentially optimal variables for predicting OS and PFS, respectively. Finally, the optimal predictors for OS encompassed age, tumor size, APRI, NLR, and PLR (Figure 3A, 3B), while those for PFS were gender, age, TNM stage, PHT, AFP, and APRI (Figure 4A, 4B).

### Development of OS and PFS model

**Figure 5:**
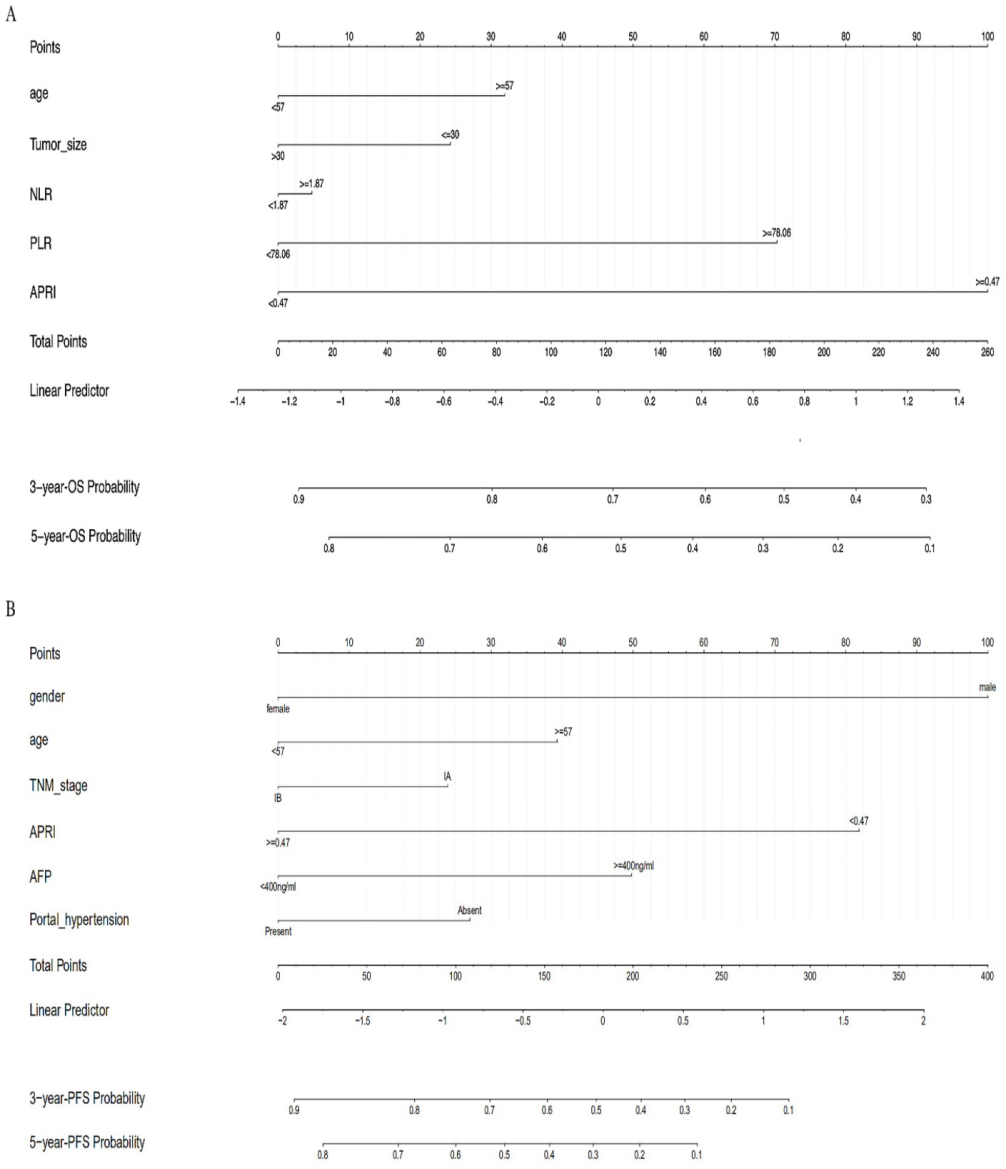
(A) The nomogram to predict OS; (B) The nomogram to predict PFS.

Multivariate COX regression analysis was performed to evaluate the proposed OS and PFS models, and the corresponding nomograms were plotted. The OS nomogram was plotted based on the variables of age, tumor size, NLR, PLR and APRI to evaluate the 3- and 5-year OS probability (Figure 5A). And the PFS nomogram was constructed to predict the 3- and 5-year PFS probability based on the following variables: gender, age, TNM stages, PHT, AFP, and APRI (Figure 5B). The multivariate COX regression analysis showed that APRI was an independent predictor for OS and PFS (Table 2).

### Apparent performance of the nomograms and Bootstrap internal validation

The AUCs of the nomograms for OS and PFS were 0.766 and 0.723, respectively. The C-indexes of the predictive nomograms were 0.738 (95% CI: 0.632-0.844) and 0.657 (95% CI: 0.538-0.777), respectively. Bootstrap internal validation (B=10000 Bootstrap resamples) showed that the nomograms for OS (C-index: 0.790, 95% CI: 0.684-0.896) and PFS (C-index: 0.739, 95% CI: 0.619-0.859) were verified to have satisfactory prognostic discriminations.

### Relationships between APRI and Survival

**Figure 6:**
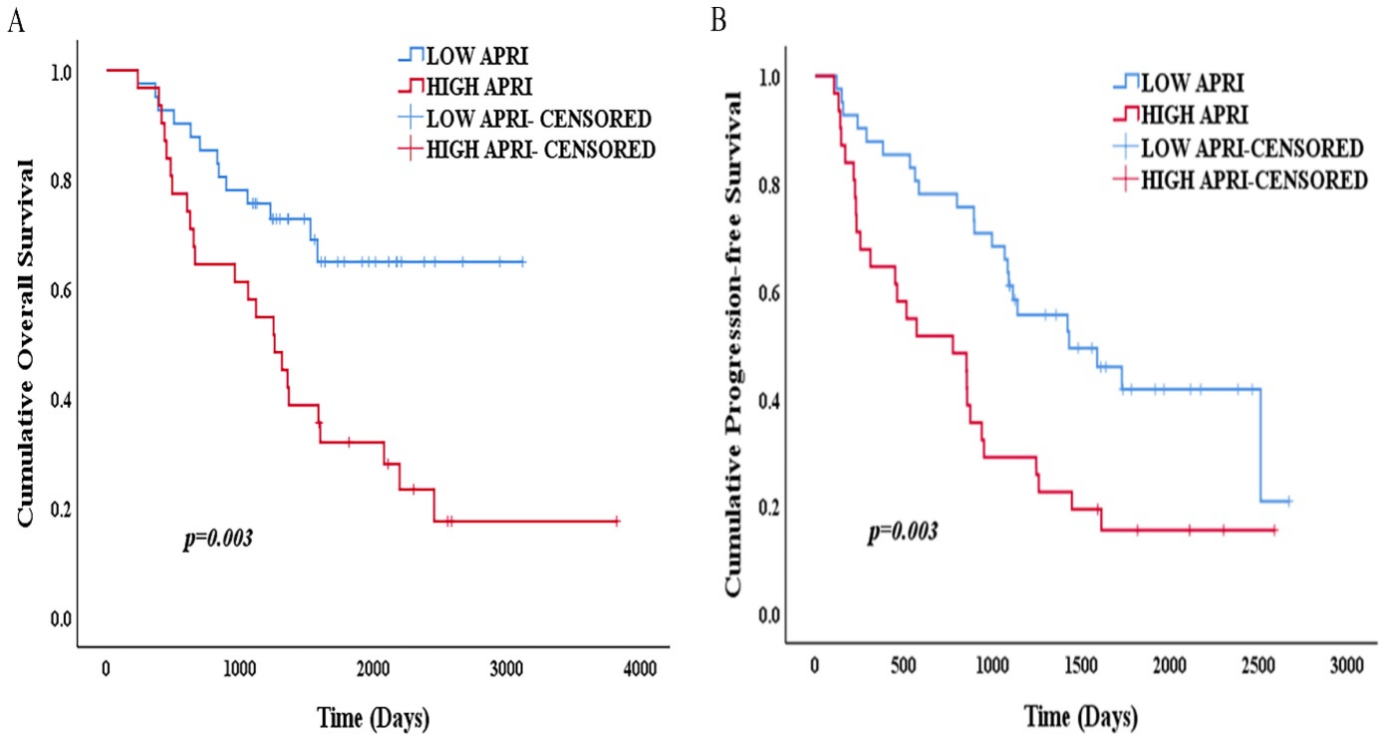
(A) Kaplan-Meier survival curve of OS according to APRI; (B) Kaplan-Meier survival curve of PFS according to APRI.

The Kaplan-Meier survival analysis and log-rank test showed that the 1-, 3- and 5-year OS rates were 97.6%, 75.6% and 64.9% in the low APRI group (<0.47) and 96.8%, 58.1% and 31.9% in the high APRI group (≥0.47) (p=0.003) (Figure 6A). The 1-, 3- and 5-year PFS rates were 87.8%, 61% and 41.7%, respectively, in the low APRI group (<0.47) and 64.5%, 29% and 15.5% in the high APRI group (≥0.47) (p=0.003) (Figure 6B). The low APRI (<0.47) group presented more favourable OS and PFS rates.

### Relationships between APRI and clinical characteristics

Spearman's rho showed that the APRI was related to the HBV status (r=0.243, *p*=0.040), Child-Turcotte-Pugh grade (r=-0.350, *p*=0.003) and portal hypertension (r=-0.521, *p*=0.000). The results showed weak correlations between APRI and HBV infection pattern and between APRI and Child-Turcotte-Pugh grade, but suggested a closely correlation between APRI and portal hypertension (Table 3).

## Discussion

As far as we know, our research is the first to explore the prognostic role of the novel inflammatory marker APRI in HBV-related small solitary HCC patients who have received SBRT by constructing APRI nomograms. At internal Bootstrap resampling validation, the user-friendly nomograms exhibited excellent discrimination abilities, showing accurate individualized prediction for 3- and 5-year OS and PFS. Our study revealed that APRI was justified as an inflammatory factor that was independently correlated with OS and PFS of HBV-associated small solitary liver cancer patients receiving SBRT.

SBRT is one of the radical treatment for early HCC that features the 3-year OS rate variably ranges from 58.6% to 73.5%^9^, ^26^. Compared with liver resection and transplantation, the research and application of SBRT in HCC as primary therapy are far less intensive and extensive. Therefore, the reason of wide variation of outcome is still unclear, which we devote to exploring. Tumor burden and liver functional reserve may partially contribute to the wide variation of outcome and highly determine the management and prognosis in HCC^27^. As to SBRT for HCC, Previous studies on SBRT for HCC have suggested that Child-Pugh classification, tumor biology and dosimetry parameters of radiotherapy may be prognostic factors of SBRT for HCC while ignoring the influence of underlying hepatitis background^8^, ^28^. Since we only account for small solitary HCC (≤5cm in size) in the study, the tumor burden have no significant influence on the prognosis, which is in accordance with prior publication^29^. Liver reserve is widely evaluated by Child-Pugh classification system, which exits the intrinsic defects for the two subjective items^30^, ^31^. And liver biopsy is the gold standard to grade hepatic fibrosis or cirrhosis while it is invasive and costly. Instead, non-invasive liver reserve markers have developed to assess liver disease over the past decades^32^. In our study, APRI-- the only one non-invasive liver reserve markers among the four serum inflammatory markers-- displayed an independent prognostic factor of OS and PFS. Low APRI (<0.47) before treatment were favourable independent prognostic factors for this patient group. In addition, our research also revealed that APRI is closely related to portal hypertension in baseline clinical characteristics, which also reflects APRI's recognition of advanced liver fibrosis and cirrhosis.

In 2003, Wai *et al.*^33^ first proposed the concept of the APRI and used it as a substitute index for liver biopsy in the non-invasive diagnosis of fibrosis and liver cirrhosis. Since then, Hung *et al.*^14^ have reported for the first time the prognostic value of APRI for HBV-associated small hepatocellular carcinoma after resection. They believe that an APRI < 0.47 indicates better recurrence-free survival (RFS) and OS and is a reliable indicator for identifying advanced fibrosis in noncancer regions. Maegawa *et al.*^34^ and Cheng *et al.*^35^ started to study the prognostic factors of complications such as perioperative and postoperative liver failure of liver cancer, strengthening and highlighting the ability of detecting APRI to evaluate liver reserve before treatment. Since then, Kao *et al.*^36^ and Zhu *et al.*^37^ have also reported the prognostic value of APRI under different intervention schemes, such as RFA and TACE. Now we involve the SBRT patients to such prognostic analysis of HCC. Although the cut-off value of APRI and the study endpoint are not exactly same, the views are consistent; that is, a low APRI predicts better overall survival and liver reserve.

Otherwise, APRI, a non-invasive liver reserve marker, is objective, accurate, simple and repeatable in comparison with Child-Pugh classification. Such continuous data obtained by calculating the AST-to-PLT ratio without upper and lower limit values expand the scope of assessments for liver cancer, which is better able to classify the severity of the disease, suggesting that APRI is more sensitive for identifying patients with small HCC who have undergone SBRT than the Child-Pugh classification. AST introduced in this parameter reveals that inflammatory responses resulting from chronic viral infections and damage to the immune system in the host are intrinsic factors that facilitate the supportive microenvironment for tumor development and influence the prognosis of liver cancer^38^. Previous studies have shown that low AST levels are associated with longer disease-free survival and a low recurrence rate after surgical therapies for liver cancer, while high AST levels represent continuous and potent invasive inflammatory responses^39^, ^40^. In this way, repeated cycles of necrosis and the regeneration of hepatocytes induced by such inflammatory responses can result in multiple recurrent lesions in the residual liver after resection^41^. Besides, platelets, an important immune surveillance mechanism of tumor cells, have been recognized to play key roles in tumor immunity and the microenvironment^42^. Platelets have also been proven to facilitate and regulate tumor angiogenesis^43^ and inhibit the biofunctions of immune cells such as NK cells through TGF-β^44^, ^45^. Therefore, a combination of AST and platelet count can reflect the prognosis of liver cancer from liver reserve, inflammatory and immune levels. That is the reason why APRI can stand out to be a promising indicator for early HCC patients. Moreover, SBRT triggers tumor cell apoptosis via the caspase-3 signalling pathway to positively regulate tumor immunity^10^, ^46^. So we speculate that the decline of platelets may be due to the effects of SBRT and the degree of decline can be associated with the prognosis of small HCC. From the original data, we have found that the platelet counts of 4 patients with high platelet counts (>300×10^9^/L) before treatment decreased to the normal range after SBRT treatment, and the OS time of these patients reached more than 3 years. Therefore, SBRT may help inhibit the adverse prognosis induced by a high PLT count, but further confirmation is still needed in subsequent studies.

Our study also analyzed prognostic effects of the other inflammatory markers NLR, PLR and LMR before treatment on variable selection and nomogram development. No significant predictive values for the OS of patients with HBV-associated small solitary HCC following SBRT were identified. By far, no consensus concerning the independent predictive values of pre-treatment NLR and PLR has been reached. Zheng *et al.* have carried out a meta-analysis and reported that an elevated pre-treatment level of NLR or PLR indicated poor outcomes for HCC patients 47. However, Kinoshita* et al.,* Zhang* et al.* and Zhuang* et al.* believed that pre-treatment NLR and PLR are not correlated to the survival of HCC 22, 48, 49. Our research identified the prognostic factors on HBV-related small solitary HCC since the small HCC patients enrolled in our study were limited to solitary nodule and HBV infection as the only etiological factor.

Our research also has some inherent limitations. This study was conducted in a single centre without external validation and involved a small sample size. Individual patients also have other medical comorbidities, such as diabetes. All of the abovementioned factors may affect our results. Therefore, the results should be interpreted cautiously and be further validated through updated sample size and extended follow-up or be confirmed in larger prospective studies.

## Conclusions

In summary, nomograms based onAPRI are prominent in predicting the OS and PFS in HBV-associated small solitary HCC patients treated with SBRT. APRI is an independent prognostic factor for both OS and PFS and could be a promising indicator that can be widely applied in clinic. More prognostic markers are needed to codetermine the potential patients benefit from SBRT as primary therapy for HCC.

## Figures and Tables

**Table 1:** Patients and disease characteristics.

Categories	Median (range)	N(%)
Sex	N/A	
Male		61(84.7%)
Female		11(15.3%)
Age (years)	57(30-84)	
≥57		37(51.4%)
<57		35(48.6%)
HBV status	N/A	
HBsAg (+)		46(63.9%)
HBcAb (+) HBeAb (+)		7(9.7%)
HBcAb (+) HBeAg (+)		19(26.4%)
HBV DNA	N/A	
Positive		36(50%)
Negative		36(50%)
CTP grade	5(5-8)	
A(Score 5/6)		63(87.5%)
B(Score 7/8/9)		9(12.5%)
Portal hypertension	N/A	
Absent		57(79.2%)
Present		15(20.8%)
Tumor size (mm)	34(15-50)	
≤30		31(43.1%)
>30, ≤50		41(56.9%)
TNM stage	N/A	
IA(T1aN0M0)		27(37.5%)
IB(T1bN0M0)		45(62.5%)
AFP (ng/ml)	48.72(0.72-1210)	
≥400		20(27.8%)
<400		52(72.2%)
NEUT (×10^9^/L)	2.86(1.06-8.04)	
≤6.3		68(94.4%)
>6.3		4(5.6%)
LYM (×10^9^/L)	1.51(0.68-3.83)	
≤3.2		68(94.4%)
>3.2		4(5.6%)
MONO (×10^9^/L)	0.456(0.19-0.93)	
≤0.6		58(80.6%)
>0.6		14(19.4%)
PLT (×10^9^/L)	160.5(31-355)	
>300		4(5.6%)
≤300		68(94.4%)
AST (U/L)	29(13-199)	
≤45		58(80.6%)
>45		14(19.4%)
APRI	0.42(0.12-14.27)	
≥0.42		36(50%)
<0.42		36(50%)
NLR	1.88(0.56-5.08)	
≥1.88		38(52.8%)
<1.88		34(47.2%)
PLR	94.36(30.39-289.86)	
≥94.36		37(51.4%)
<94.36		35(48.6%)
LMR	3.485(1.4-6.94)	
≥3.485		37(51.4%)
<3.485		35(48.6%)
Radiotherapy		
Regimen	N/A	
36-48 Gy/3 fractions		54(75%)
40-48 Gy/4 fractions		15(20.8%)
45-48 Gy/5 fractions		3(4.2%)
BED10 (Gy)	100.8(79.2-124.8)	
≥100		43(59.7%)
<100		29(40.3%)

**Abbreviations**: NA: Not applicable; HBV DNA: hepatis B virus deoxyribonucleic acid; AFP: alpha fetoprotein; CTP: Child-Turcotte-Pugh; NEUT: neutrophil; LYM: lymphocyte; MONO: monocyte; PLT: platelet; AST: aspartate aminotransferase; APRI: aspartate aminotransferase-to-platelet index; NLR: neutrophil-to-lymphocyte ratio; PLR: platelet-to-lymphocyte ratio; LMR: lymphocyte-to-monocyte ratio; BED: biological effective dose; TNM : American Joint Committee on Cancer (AJCC) Tumor-Node-Metastasis Staging for Hepatocellular Cancer (8th ed., 2017).

**Table 2:** Multivariate COX regression analysis for OS and PFS

Period	Variable	Multivariate COX analysis				
		Coef	SE	Wald Z	HR (95% CI)	*P*
OS	Age (<57 vs ≥57)	0.3385	0.0167	-1.65	1.401(0.499-2.809)	0.342
	Tumor size (≤30 vs >30, ≤50)	-0.2572	0.3559	-0.22	0.790(0.391-1.597)	0.512
	NLR (≥1.87vs<1.87)	0.0503	0.1852	0.90	1.074(0.539-2.143)	0.838
	PLR (≥78.06 vs <78.06)	0.7448	0.0053	-0.41	1.917(0.761-4.828)	0.167
	APRI (≥0.47 vs <0.47)	1.0595	0.4377	1.74	3.060(1.378-6.795)	0.006
PFS	Gender(Male vs Female)	-1.1067	0.5322	-2.08	0.331(0.116-0.938)	0.038
	Age (<57 vs ≥57)	0.4349	0.3104	1.40	1.545(0.841-2.839)	0.161
	TNM stage (IA vs IB)	-0.2640	0.3027	-0.87	0.770(0.424-1.390)	0.383
	APRI (<0.47 vs≥0.47)	-0.9058	0.3657	-2.48	0.404(0.197-0.828)	0.013
	AFP (≥400 vs <400)	0.5510	0.3474	1.59	1.735(0.878-3.428)	0.113
	PHT (Absent vs Present)	-0.3000	0.4264	-0.70	0.742(0.322-1.711)	0.484

**Abbreviations**: Coef, regression coefficient; SE, standard error; HR, hazard ratio; CI, confidential intervals; NLR, neutrophil-to-lymphocyte ratio; PLR, platelet-to-lymphocyte ratio; APRI, aspartate aminotransferase-to-platelet index; PHT, Portal hypertension; TNM, American Joint Committee on Cancer (AJCC) Tumor-Node-Metastasis Staging for Hepatocellular Cancer (8th ed., 2017).

**Table 3:** Relationships between APRI and clinical characteristics before SBRT

Categories	APRI≥0.47 N =31	APRI<0.47 N =41	Coefficient (r)	*p*
Sex				
Male	27	34	0.057	0.115
Female	4	7		
Age (years)				
≥57	12	25	-0.221	0.063
<57	19	16		
HBV status				
HBsAg(+)	24	22	0.243	0.040
HBcAb(+)HBeAb(+)	2	5		
HBcAb(+)HBeAg(+)	5	14		
HBV DNA				
Positive	12	18	-0.052	0.663
Negative	19	23		
CTP grade				
A	23	40	-0.350	0.003
B	8	1		
Portal hypertension				
Absent	17	40	-0.521	0.000
Present	14	1		
Tumor size (mm)				
≤30	15	16	0.094	0.434
>30, ≤50	16	25		
TNM stage				
IA	12	15	0.022	0.856
IB	19	26		
AFP (ng/ml)				
≥400	8	12	0.038	0.750
<400	23	29		
Radiotherapy				
Regimen				
36-48 Gy/3 fractions	22	32	-0.089	0.459
45-48 Gy/4 fractions	7	8		
45-48 Gy/5 fractions	2	1		
BED10 (Gy)				
≥100	15	27	-0.175	0.140
<100	16	14		

**Abbreviations**: HBV DNA: hepatis B virus deoxyribonucleic acid; AFP: alpha fetoprotein; CTP: Child-Turcotte-Pugh; APRI: aspartate aminotransferase-to-platelet index; BED: biological effective dose; TNM: American Joint Committee on Cancer (AJCC) TNM Staging for Hepatocellular Cancer (8th ed., 2017).

## References

[1] Bray F, Ferlay J, Soerjomataram I, Siegel RL, Torre LA, Jemal A (2018). Global cancer statistics 2018: GLOBOCAN estimates of incidence and mortality worldwide for 36 cancers in 185 countries. CA Cancer J Clin.

[2] Wu B, Zhou J, Ling G, Zhu D, Long Q (2018). CalliSpheres drug-eluting beads versus lipiodol transarterial chemoembolization in the treatment of hepatocellular carcinoma: a short-term efficacy and safety study. World journal of surgical oncology.

[3] Villanueva A (2019). Hepatocellular Carcinoma. N Engl J Med.

[4] Llovet J, Ducreux M, Lencioni R, Di Bisceglie A, Galle P, Dufour J (2012). European Association for the Study of the Liver European Organisation for Research and Treatment of Cancer: EASL-EORTC clinical practice guidelines: management of hepatocellular carcinoma. J Hepatol.

[5] Zhang T, Sun J, He W, Li H, Piao J, Xu H (2018). Stereotactic body radiation therapy as an effective and safe treatment for small hepatocellular carcinoma. BMC Cancer.

[6] Zeng ZC, Seong J, Yoon SM, Cheng JC, Lam KO, Lee AS (2017). Consensus on Stereotactic Body Radiation Therapy for Small-Sized Hepatocellular Carcinoma at the 7th Asia-Pacific Primary Liver Cancer Expert Meeting. Liver Cancer.

[7] Benson AB, D'Angelica MI, Abbott DE, Abrams TA, Alberts SR, Anaya DA (2019). Guidelines Insights: Hepatobiliary Cancers, Version 2.2019. J Natl Compr Canc Netw.

[8] Su TS, Liang P, Liang J, Lu HZ, Jiang HY, Cheng T (2017). Long-Term Survival Analysis of Stereotactic Ablative Radiotherapy Versus Liver Resection for Small Hepatocellular Carcinoma. Int J Radiat Oncol Biol Phys.

[9] Su TS, Liang P, Lu HZ, Liang J, Gao YC, Zhou Y (2016). Stereotactic body radiation therapy for small primary or recurrent hepatocellular carcinoma in 132 Chinese patients. J Surg Oncol.

[10] Luo H, Ge H, Cui Y, Zhang J, Fan R, Zheng A (2018). Systemic Inflammation Biomarkers Predict Survival in Patients of Early Stage Non-Small Cell Lung Cancer Treated With Stereotactic Ablative Radiotherapy - A Single Center Experience. J Cancer.

[11] Wang FS, Fan JG, Zhang Z, Gao B, Wang HY (2014). The global burden of liver disease: the major impact of China. Hepatology.

[12] Stern MC, Umbach DM, Yu MC, London SJ, Zhang ZQ, Taylor JA (2001). Hepatitis B, aflatoxin B(1), and p53 codon 249 mutation in hepatocellular carcinomas from Guangxi, People's Republic of China, and a meta-analysis of existing studies. Cancer Epidemiol Biomarkers Prev.

[13] Yang JD, Kim WR, Coelho R, Mettler TA, Benson JT, Sanderson SO (2011). Cirrhosis is present in most patients with hepatitis B and hepatocellular carcinoma. Clin Gastroenterol Hepatol.

[14] Hung HH, Su CW, Lai CR, Chau GY, Chan CC, Huang YH (2010). Fibrosis and AST to platelet ratio index predict post-operative prognosis for solitary small hepatitis B-related hepatocellular carcinoma. Hepatol Int.

[15] Yang R, Chang Q, Meng X, Gao N, Wang W (2018). Prognostic value of Systemic immune-inflammation index in cancer: A meta-analysis. J Cancer.

[16] Matsumoto M, Wakiyama S, Shiba H, Haruki K, Futagawa Y, Ishida Y (2018). Usefulness of aspartate aminotransferase to platelet ratio index as a prognostic factor following hepatic resection for hepatocellular carcinoma. Mol Clin Oncol.

[17] Wang C, He W, Yuan Y, Zhang Y, Li K, Zou R (2020). Comparison of the prognostic value of inflammation-based scores in early recurrent hepatocellular carcinoma after hepatectomy. Liver Int.

[18] Mano Y, Yoshizumi T, Yugawa K, Ohira M, Motomura T, Toshima T (2018). Lymphocyte-to-Monocyte Ratio Is a Predictor of Survival After Liver Transplantation for Hepatocellular Carcinoma. Liver Transpl.

[19] Sprinzl MF, Kirstein MM, Koch S, Seib ML, Weinmann-Menke J, Lang H (2019). Improved Prediction of Survival by a Risk Factor-Integrating Inflammatory Score in Sorafenib-Treated Hepatocellular Carcinoma. Liver Cancer.

[20] Shen Y, Wang H, Chen X, Li W, Chen J (2019). Prognostic significance of lymphocyte-to-monocyte ratio and platelet-to-lymphocyte ratio in patients with hepatocellular carcinoma undergoing transcatheter arterial chemoembolization and radiofrequency ablation. Onco Targets Ther.

[21] D'Emic N, Engelman A, Molitoris J, Hanlon A, Sharma NK, Moeslein FM (2016). Prognostic significance of neutrophil-lymphocyte ratio and platelet-lymphocyte ratio in patients treated with selective internal radiation therapy. Journal of gastrointestinal oncology.

[22] Zhuang Y, Yuan B-Y, Hu Y, Chen G-W, Zhang L, Zhao X-M (2019). Pre/Post-Treatment Dynamic of Inflammatory Markers Has Prognostic Value in Patients with Small Hepatocellular Carcinoma Managed by Stereotactic Body Radiation Therapy. Cancer management and research.

[23] EASL-EORTC clinical practice guidelines (2012). management of hepatocellular carcinoma. J Hepatol.

[24] Bruix J, Sherman M (2011). Management of hepatocellular carcinoma: an update. Hepatology.

[25] Jones B, Dale R, Deehan C, Hopkins K, Morgan D (2001). The role of biologically effective dose (BED) in clinical oncology. Clinical oncology.

[26] Kwon JH, Bae SH, Kim JY, Choi BO, Jang HS, Jang JW (2010). Long-term effect of stereotactic body radiation therapy for primary hepatocellular carcinoma ineligible for local ablation therapy or surgical resection. Stereotactic radiotherapy for liver cancer. BMC Cancer.

[27] Ho S-Y, Liu P-H, Hsu C-Y, Hsia C-Y, Lee Y-H, Lee R-C (2017). Prognostic role of noninvasive liver reserve markers in patients with hepatocellular carcinoma undergoing transarterial chemoembolization. PloS one.

[28] Sun J, Zhang T, Wang J, Li W, Zhang A, He W (2019). Biologically effective dose (BED) of stereotactic body radiation therapy (SBRT) was an important factor of therapeutic efficacy in patients with hepatocellular carcinoma (</=5 cm). BMC Cancer.

[29] Kuo HT, Que J, Lin LC, Yang CC, Koay LB, Lin CH (2017). Impact of tumor size on outcome after stereotactic body radiation therapy for inoperable hepatocellular carcinoma. Medicine (Baltimore).

[30] Johnson PJ, Berhane S, Kagebayashi C, Satomura S, Teng M, Reeves HL (2015). Assessment of liver function in patients with hepatocellular carcinoma: a new evidence-based approach-the ALBI grade. J Clin Oncol.

[31] Durand F, Valla D (2005). Assessment of the prognosis of cirrhosis: Child-Pugh versus MELD. J Hepatol.

[32] Castera L (2012). Noninvasive methods to assess liver disease in patients with hepatitis B or C. Gastroenterology.

[33] Wai CT, Greenson JK, Fontana RJ, Kalbfleisch JD, Marrero JA, Conjeevaram HS (2003). A simple noninvasive index can predict both significant fibrosis and cirrhosis in patients with chronic hepatitis C. Hepatology.

[34] Maegawa FB, Shehorn L, Aziz H, Kettelle J, Jie T, Riall TS (2019). Association Between Noninvasive Fibrosis Markers and Postoperative Mortality After Hepatectomy for Hepatocellular Carcinoma. JAMA Netw Open.

[35] Cheng J, Zhao P, Liu J, Liu X, Wu X (2016). Preoperative aspartate aminotransferase-to-platelet ratio index (APRI) is a predictor on postoperative outcomes of hepatocellular carcinoma. Medicine (Baltimore).

[36] Kao WY, Chiou YY, Hung HH, Chou YH, Su CW, Wu JC (2011). Risk factors for long-term prognosis in hepatocellular carcinoma after radiofrequency ablation therapy: the clinical implication of aspartate aminotransferase-platelet ratio index. Eur J Gastroenterol Hepatol.

[37] Zhu GQ, Wang K, Wang B, Zhou YJ, Yang Y, Chen EB (2019). Aspartate aminotransferase-to-platelet ratio index predicts prognosis of hepatocellular carcinoma after postoperative adjuvant transarterial chemoembolization. Cancer Manag Res.

[38] Chen L, Zhang Q, Chang W, Du Y, Zhang H, Cao G (2012). Viral and host inflammation-related factors that can predict the prognosis of hepatocellular carcinoma. Eur J Cancer.

[39] Ercolani G, Grazi GL, Ravaioli M, Del Gaudio M, Gardini A, Cescon M (2003). Liver resection for hepatocellular carcinoma on cirrhosis: univariate and multivariate analysis of risk factors for intrahepatic recurrence. Ann Surg.

[40] Witjes CD, JN IJ, van der Eijk AA, Hansen BE, Verhoef C, de Man RA (2011). Quantitative HBV DNA and AST are strong predictors for survival after HCC detection in chronic HBV patients. Neth J Med.

[41] Ko S, Nakajima Y, Kanehiro H, Hisanaga M, Aomatsu Y, Kin T (1996). Significant influence of accompanying chronic hepatitis status on recurrence of hepatocellular carcinoma after hepatectomy. Result of multivariate analysis. Ann Surg.

[42] Placke T, Orgel M, Schaller M, Jung G, Rammensee HG, Kopp HG (2012). Platelet-derived MHC class I confers a pseudonormal phenotype to cancer cells that subverts the antitumor reactivity of natural killer immune cells. Cancer Res.

[43] Ho-Tin-Noe B, Carbo C, Demers M, Cifuni SM, Goerge T, Wagner DD (2009). Innate immune cells induce hemorrhage in tumors during thrombocytopenia. Am J Pathol.

[44] Rachidi S, Metelli A, Riesenberg B, Wu BX, Nelson MH, Wallace C (2017). Platelets subvert T cell immunity against cancer via GARP-TGFbeta axis. Sci Immunol.

[45] Kopp HG, Placke T, Salih HR (2009). Platelet-derived transforming growth factor-beta down-regulates NKG2D thereby inhibiting natural killer cell antitumor reactivity. Cancer Res.

[46] Meng MB, Wang HH, Cui YL, Wu ZQ, Shi YY, Zaorsky NG (2016). Necroptosis in tumorigenesis, activation of anti-tumor immunity, and cancer therapy. Oncotarget.

[47] Zheng J, Cai J, Li H, Zeng K, He L, Fu H (2017). Neutrophil to Lymphocyte Ratio and Platelet to Lymphocyte Ratio as Prognostic Predictors for Hepatocellular Carcinoma Patients with Various Treatments: a Meta-Analysis and Systematic Review. Cellular physiology and biochemistry: international journal of experimental cellular physiology, biochemistry, and pharmacology.

[48] Zhang T, Liu Z, Zhao X, Mao Z, Bai L (2019). A novel prognostic score model based on combining systemic and hepatic inflammation markers in the prognosis of HBV-associated hepatocellular carcinoma patients. Artificial cells, nanomedicine, and biotechnology.

[49] Kinoshita A, Onoda H, Imai N, Iwaku A, Oishi M, Fushiya N (2012). Comparison of the prognostic value of inflammation-based prognostic scores in patients with hepatocellular carcinoma. British journal of cancer.

